# An Objective Approach for *Burkholderia pseudomallei* Strain Selection as Challenge Material for Medical Countermeasures Efficacy Testing

**DOI:** 10.3389/fcimb.2012.00120

**Published:** 2012-09-26

**Authors:** Kristopher E. Van Zandt, Apichai Tuanyok, Paul S. Keim, Richard L. Warren, H. Carl Gelhaus

**Affiliations:** ^1^Battelle, ColumbusOH, USA; ^2^Center for Microbial Genetics and Genomics, Northern Arizona UniversityFlagstaff, AZ, USA

**Keywords:** *pseudomallei*, aerosol, biodefense, animal models, virulence, genome, sequencing, passage history

## Abstract

*Burkholderia pseudomallei* is the causative agent of melioidosis, a rare disease of biodefense concern with high mortality and extreme difficulty in treatment. No human vaccines are available that protect against *B. pseudomallei* infection, and with the current limitations of antibiotic treatment, the development of new preventative and therapeutic interventions is crucial. Although clinical trials could be used to test the efficacy of new medical countermeasures (MCMs), the high mortality rates associated with melioidosis raises significant ethical issues concerning treating individuals with new compounds with unknown efficacies. The US Food and Drug Administration (FDA) has formulated a set of guidelines for the licensure of new MCMs to treat diseases in which it would be unethical to test the efficacy of these drugs in humans. The FDA “Animal Rule” 21 CFR 314 calls for consistent, well-characterized *B. pseudomallei* strains to be used as challenge material in animal models. In order to facilitate the efficacy testing of new MCMs for melioidosis using animal models, we intend to develop a well-characterized panel of strains for use. This panel will comprise of strains that were isolated from human cases, have a low passage history, are virulent in animal models, and are well-characterized phenotypically and genotypically. We have reviewed published and unpublished data on various *B. pseudomallei* strains to establish an objective method for selecting the strains to be included in the panel of *B. pseudomallei* strains with attention to five categories: animal infection models, genetic characterization, clinical and passage history, and availability of the strain to the research community. We identified 109 strains with data in at least one of the five categories, scored each strain based on the gathered data and identified six strains as candidate for a *B. pseudomallei* strain panel.

## Introduction

*Burkholderia pseudomallei* is a Gram-negative bacterial pathogen responsible for the disease melioidosis, which is endemic in Southeast Asia, and northern Australia. *B. pseudomallei* can be cultured from tropical soils without requiring a host for environmental persistence (Kaestli et al., 2009; Currie et al., 2010). Melioidosis is historically associated with a high mortality rate due to the speed with which septicemia develops and the inherent resistance of the bacteria to several classes of antibiotics. While the overall mortality rate of melioidosis is 50% in Thailand, it is only 19% in the endemic areas of Australia (Limmathurotsakul et al., 2010). The difference between the mortality rates is most likely a result of the differing availability of efficacious treatment, though pathogen strain and host population differences may play a role. Although prolonged courses of antibiotic treatment (at least 10 days of intravenous antibiotics, followed by 12–20 weeks of oral antibiotics) are recommended, recurrent disease is common (at a rate of ≥6% in the first year; Chaowagul et al., 1993; Currie et al., 2000).

In addition to naturally occurring infections, *B. pseudomallei* is considered a biodefense threat because of the ease of acquiring strains from the environment, the ability to genetically manipulate the agent to be multiply antibiotic resistant, and the lack of a melioidosis vaccine (Vietri and Deshazer, 2007). Given the lengthy treatment with suboptimal outcomes, there is significant interest in developing improved medical countermeasures (MCMs) against melioidosis. In addition to improved antibiotic treatments, licensed vaccines are not currently available for melioidosis prophylaxis. In the case of *B. pseudomallei*, the limited number of confirmed respiratory acquired melioidosis cases each year call into question the feasibility of clinical trials for proper evaluation of therapeutics and/or vaccines. Experimental infection of humans with *B. pseudomallei* creates ethical concerns because therapeutic options are limited and consist of lengthy regimens. If melioidosis animal models are to be used to license new MCMs under the Food and Drug Administration (FDA) Animal Rule, well-characterized *B. pseudomallei* sources will be needed by the testing community. Since a number of different *B. pseudomallei* strains have been used in a variety of animal models, comparisons of the data are difficult. We reviewed the publicly available literature and used personal communications to gather data relevant to strain source, passage history, virulence, genotype, phenotype, and availability of *B. pseudomallei* strains with focus on those. Additionally, attributes of *B. pseudomallei* strains which apply to the FDA Animal Rule, particularly, animal infection models, genetic characterization, clinical and passage history, and the availability of strains to the research community were considered. FDA Animal Rule draft guidance from 2009 states “the challenge agent used in animal studies generally should be identical to the etiologic agent that causes the human disease (FDA, 2009).” For melioidosis, the agent is *B. pseudomallei*, but many different strains have been isolated from human cases. The genetic plasticity of *B. pseudomallei* dictates that strains with well-documented and low numbers of passages are preferred (Holden et al., 2004). Further, *B. pseudomallei* is known to have an unstable genome and having a reference genome sequence is desirable to monitor any genetic mutation and drift that may occur from laboratory passages. The FDA Animal Rule draft guidance also states “The disease manifestations, including the outcome (morbidity or mortality), should be compared between untreated animals and untreated humans (FDA, 2009).” Since melioidosis is a lethal disease, we also examined lethality in the animal model literature. Finally, as the development of animal models and MCMs will be an effort spanning multiple laboratories, we were interested in those *B. pseudomallei* strains that had the fewest impediments to wide distribution, such as licenses or restrictive material transfer agreements. We developed a scoring matrix to select six strains for inclusion in a panel to be tested in animal model development to meet the criteria of the FDA Animal Rule. Ideally, bacterial challenge material used in efficacy studies under the FDA Animal Rule would be a strain of *B. pseudomallei* that was recently isolated from a lethal human case of inhalational melioidosis, possess limited passage, exhibit virulence in one or more animal model of melioidosis, be well-characterized, and be freely available to laboratories licensed to work with *B. pseudomallei*. Using these and other criteria, a ranking system was developed and used to identify strains that will be included in a *B. pseudomallei* reference panel. Each section of this review will focus on *B. pseudomallei* strains that had the highest score for a single criterion, based on the availability of information for that criterion.

## Isolation and Passage History

We had two criteria associated with the strain history; the patient history associated with the isolate and documented laboratory passage history. For isolation, strains were assigned a score on a scale of zero through five using the following six criteria:

0 points for no data on isolation
1 point for environmental isolate
2 points for isolation from an animal infection
3 points for isolation from a human clinical case, with no other data available
4 points for isolation from a human clinical case without pulmonary presentation
5 points for isolation from a human case with pulmonary presentation.

Using the above scoring matrix, three strains were assigned the highest score of 5 (isolated from an acute human case with pulmonary presentation, Table 1). Strain S141 was isolated in Ubon Ratchathani, NE, Thailand from a 73-year-old male patient with pneumonia. Strain 1710a was isolated from the blood culture in 1996 from a 52-year-old male rice farmer with a new diagnosis of diabetes mellitus presenting to Sappasithiprasong Hospital, Ubon Ratchathani, NE, Thailand (http://pathema.Jcvi.org/cgi-bin/Burkholderia/shared/HtmlPage.cgi?page=strains). The patient had disseminated disease, including bacteremia, lung involvement, and soft tissue involvement. The patient survived to discharge, but was readmitted in 1999 and died 1 day after readmission (http://pathema.jcvi.org/cgi-bin/Burkholderia/shared/HtmlPage.cgi?page=strains). Strain 406e was isolated from a toe swab in 1988 from a 21-year-old male laborer presenting to Sappasithiprasong Hospital (http://pathema.jcvi.org/cgi-bin/Burkholderia/shared/HtmlPage.cgi?page=strains). The patient had disseminated disease including bacteremia, lung, skin, and renal tract involvement. The patient succumbed to infection on the second day of admission (http://pathema.jcvi.org/cgi-bin/Burkholderia/shared/HtmlPage.cgi?page=strains; M. Wolcott, personal communication).

**Table 1:** ***Burkholderia pseudomallei* strains with well described clinical isolations**.

Bacterial strain	Date of isolation	Country of origin	Clinical history	Isolation score
1710a	1996	Thailand	52-Year-old male rice farmer presenting to Sappasithiprasong Hospital, Ubon Ratchathani, NE, Thailand	5
S141	Unknown	Thailand	Isolated in Ubon Thailand from a 73-year-old male patient with pneumonia	5
406e	1988	Thailand	Toe swab isolated from a 21-year-old male laborer with disseminated disease including bacteremia, lung, skin, and renal tract involvement	5
MSHR305	Unknown	Australia	This strain was isolated from an autopsy sample from the brain of a fatal melioidosis encephalomyelitis case at the Royal Darwin Hospital, Northern Territory, Australia	4
1106a	1993	Thailand	23-Year-old female rice farmer presenting to Sappasithiprasong Hospital	4
1106b	1996	Thailand	Pus aspirated from liver abscess in 1996 from patient described above (strain 1106a)	4
MSHR1655	2003	Australia	Menzies School of Health Research. Sputum isolate from a 64-year-old Darwin female patient treated at Royal Darwin Hospital	4
1710b	1999	Thailand	Blood culture in march 1999 from patient described above (strain 1710a)	4
576a	1989	Thailand	Blood culture from a 50-year-old female rice farmer presenting to Sappasithiprasong Hospital	4
B7210	1970	Australia	Isolated from empyema drain in human in Australia	4
DL2	2006	Thailand	Isolated from a fatal sepsis melioidosis case in Ubon, Thailand	4
DL25	2006	Thailand	Isolated from a fatal sepsis melioidosis case in Ubon, Thailand	4
DL28	2006	Thailand	Isolated from a fatal sepsis melioidosis case in Ubon, Thailand	4
DL35	2006	Thailand	Isolated from a sepsis melioidosis case in Ubon, Thailand. Patient survived	4
DL36	2006	Thailand	Isolated from a fatal sepsis melioidosis case in Ubon, Thailand	4
K96243	1996	Thailand	Isolated from a 34-year-old female diabetic patient at Khon Kaen Hospital, northeast Thailand with a clinical history of short incubation, septicemic infection, and rapid progression to death	4
MSHR487	Unknown	Australia	Isolated from a chronic melioidosis case. The patient was not very sick at all for several years	4
MSHR668	1995	Australia	From Menzies School of Health Research. Blood culture isolate from a 53-year-old male patient from Darwin with severe melioidosis encephalomyelitis	4
MSHR730	Unknown	Australia	Isolated from a melioidosis patient who had a chronic lung disease. The patient was not very sick at all and got better quickly	4
MSHR840	1999	Australia	Isolated from a fatal sepsis melioidosis case with risk factors. The patient lived in Queensland, Australia	4
PB08298010	2009	USA	Isolated from a severe melioidosis case in SE Arizona; patient had no foreign travel history	4
S191	Unknown	Thailand	Isolated from a 4-year-old, male, melioidosis patient who had parotid disease; patient survived	4
S241	Unknown	Thailand	Isolated from a 60-year-old, male, melioidosis patient who had parotid disease; patient survived	4
S90	Unknown	Thailand	Isolated from 1-month-old female infant who had septicemia and CNS involvement. The patient was survived to discharge	4
1026b	1993	Thailand	Blood from 29-year-old female rice farmer with diabetes mellitus and disseminated disease including bacteremia, soft tissue, joint, and splenic involvement; survived to discharge	4

With only three strains receiving the highest score, other strains that did not score as highly are included because of other qualities making them attractive for animal model research. Strain K96243 was isolated from a 34-year-old female diabetic patient in Thailand. Strain 1106a was isolated from a 23-year-old female rice farmer in Thailand in 1996. Strain 1106b was isolated from the liver abscess in 1996 from the same rice farmer from which strain 1106a was isolated. Strain MSHR305 was isolated from an autopsy sample from the brain of a melioidosis encephalomyelitis case at the Royal Darwin Hospital, Northern Territory, Australia (http://pathema.jcvi.org/cgi-bin/Burkholderia/shared/HtmlPage.cgi?page=strains). Strain MSHR668 was isolated from the blood culture of a 53-year-old male patient with severe melioidosis encephalomyelitis at the Royal Darwin Hospital in 1995. The patient required prolonged ventilation, but survived. Strain 1026b was isolated from blood culture in 1993 from 29-year-old female rice farmer with a known diagnosis of diabetes mellitus presenting to Sappasithiprasong Hospital. The patient had disseminated disease including bacteremia, soft tissue, joint, and splenic involvement (http://pathema.jcvi.org/cgi-bin/Burkholderia/shared/HtmlPage.cgi?page=strains).

The second criterion is passage history. Strains with the lowest number of passages from clinical isolation were given the highest scores. Due to the genetic plasticity of *B. pseudomallei*, passage on artificial media or through animals could readily select for genetic variants with unknown impact on pathogenicity (Ulett et al., 2001), complicating data comparisons from studies using different sources of *B. pseudomallei* with different or unknown passage histories. Therefore, available *B. pseudomallei* strains were assigned scores based on the number of passages from isolation. High scoring *B. pseudomallei* strains are shown in Table 2. Strains were assigned a score on a scale of zero through five using the following six criteria:

0 points for no passage data
1 point for >11 passages since isolation
2 points for 9–10 passages since isolation
3 points for 7–8 passages since isolation
4 points for 4–6 passages since isolation
5 points for 3 or fewer passages since isolation.

**Table 2:** ***Burkholderia pseudomallei* strains that may be obtained with low passage number**.

Bacterial strain	Passages from isolation	Minimum passage score
DL2	6	4
DL25	6	4
DL28	6	4
DL35	6	4
DL36	6	4
INT2-BP127	6	4
INT2-BP190	6	4
INT2-BP24	6	4
INT2-BP270	6	4
INT2-BP38	6	4
INT2-BP91	6	4
INT4-BP18	6	4
MSHR296	6	4
MSHR305	6	4
MSHR487	6	4
MSHR503	6	4
MSHR520	6	4
MSHR668	6	4
MSHR730	6	4
MSHR840	6	4
NAU14B6	6	4
NAU21B9	6	4
NAU24B3	6	4
NAU33A4	6	4
NAU44A6	6	4
PB08298010	4	4
RF43-BP22	6	4
RF67-BP1	6	4
RF6-BP15	6	4
RF85-BP37	6	4
S141	6	4
S191	6	4
S241	6	4
S43	6	4
S90	6	4
INT2-BP48	6	4

No strain was identified with 3 or fewer passages from isolation, although strains passaged as few as 6 times were identified.

## Animal Infection Models

Strains selected for this reference panel should have a low lethal dose with aerosol delivery in animal models for testing biodefense MCMs. As mentioned above, the ideal etiologic agent for an FDA Animal Rule study will cause lethality in both animals and humans in an acute manner. For the purposes of this review, we considered acute lethality to be death within 28 days of infection. Therefore, we examined published literature for reports of *B. pseudomallei* strains being used to experimentally infect animals. The strain reference panel is to be used to test the efficacy of MCMs against inhalational melioidosis models, so emphasis was given to strains shown to be virulent in inhalation animal models. Strains were assigned a score on a scale of zero through five using the following six criteria:

0 points for no data on animal infection
1 point for infection of animal cell cultures
2 points for animal infection indicated but no route of infection reported
3 points for acute lethality in one or more mammals via a route not intranasal (IN) or aerosol
4 points for acute lethality in one mammal with IN or aerosol route
5 points for acute lethality in multiple mammals via IN or aerosol route.

Strains 1026b, DD503, 103-67, W294, and K96423 received the highest score in this rating system (Table 3) and are discussed below.

**Table 3:** ***Burkholderia pseudomallei* strains with animal models of aerosol infection**.

Bacterial strain	LD_50_ (CFU)	Animal model	Reported animal virulence data score
1026b	10	Mouse	5
	27	Mouse	
	2.8 × 10^3^	Mouse	
DD503	1 × 10^3^	Mouse	5
	1.47 × 10^3^	Mouse	
103-67	16	Mouse	5
	16	Hamster	
W294	70	Hamster	5
K96243	5	Mouse	5
	<10; precise LD_50_ not determined	Marmoset	

### Strain 1026b

There is substantial literature on the virulence of *B. pseudomallei* 1026b in animal models of melioidosis. In murine models of pneumonic melioidosis, Jeddeloh et al. (2003) exposed BALB/c and C57BL/6 mice to whole-body aerosols. The LD_50_ of *B. pseudomallei* 1026b via this aerosol challenge method in BALB/c and C57BL/6 mice was 10 ± 8 and 27 ± 20 colony-forming units (CFU), respectively. The results suggest that both mouse strains have similar susceptibilities to the aerosolized *B. pseudomallei*. However, previous studies showed that the LD_50_ of IN challenge (an alternative to aerosol challenge) with 1026b was 2 logs higher in the resistant C57BL6 mouse when compared with the sensitive BALB/c mouse (Liu et al., 2002). Using a nose-only inhalational challenge system we showed that the LD_50_ in BALB/c mice was 2,772 CFUs (Sanford et al., 2010). Goodyear et al. (2009) reported that the LD_50_ of an IN challenge in BALB/c mice was 1 × 10^3^ CFUs. These results suggest that *B. pseudomallei* 1026b has an LD_50_ between 10 and 2,772 CFUs, depending on the route of infection and strain of mouse infected. The difference in the LD_50_ for inhalational melioidosis in BALB/c mice could reflect differences in the method of aerosol challenge, whole-body, IN, or nose-only and/or differences in the virulence of the 1026b strains used in each of these studies and may be of interest in future investigations.

### Strain DD503

Strain DD503 (Δ*amrR*-*oprA*) is a multi-drug efflux mutant strain derived from 1026b, which is hypersensitive to aminoglycosides and macrolides (Moore et al., 1999). In a whole-body inhalation BALB/c mouse model, the LD_50_ was determined to be 1 × 10^3^ CFUs. Jeddeloh et al. (2003) reported the LD_50_ of strain DD503 to be 1,467 ± 301 CFUs in BALB/c mice following whole-body aerosol exposure. It is interesting that the mouse LD_50_ of DD503 is 100 times higher than 1026b. The reduced virulence of DD503 appears to be due to a mutation in the rpsL gene (Jeddeloh et al., 2003).

### Strain 103-67

Dannenberg et al. (1958) challenged Syrian golden hamsters with an inhaled dose 3–600 LD_50_S (50–10,000 CFUs), and the inhalational LD_50_ of *B. pseudomallei* 103-67 in the hamster model appears to be approximately 17 CFUs. The lethality of inhaled *B. pseudomallei* strain 103-67 in albino mice was also examined, and in mice that succumbed to infection with an inhaled does of 18–600 LD_50_s (300–10,000 CFUs), the inhalational LD_50_ in mice of approximately 17 CFUs.

### Strain W294

Miller et al. (1948) determined the virulence of IP challenges of *B. pseudomallei* strain W294 in hamsters, guinea pigs, ferrets, rabbits, mice, rats, and non-human primates. However, the aerosol LD_50_ of W294 was determined to be 70 CFU in the hamster. Black and white mice were infected with *B. pseudomallei* W294 and 60–75% of the animals died, but the LD_50_ was not evaluated.

### Strain K96243

The vast majority of animal work done with strain K96243 was performed in mice (C57Bl/6 and BALB/c). In an effort to examine the differential susceptibility to infection between the two most commonly used mouse strains (C57Bl/6 and BALB/c), Tan et al. (2008) infected both mouse strains via various routes including the IN (nose-only aerosol). The calculated IN LD_50_ was 226 CFU in BALB/c mice and 1.5 × 10^4^ CFU in C57Bl/6 mice. Their data indicates that BALB/c mice are significantly more susceptible to infection with *B. pseudomallei* than the C57Bl/6 strain.

While large animal models of infection with *B. pseudomallei* have not yet been extensively examined, one recent study by Nelson et al. (2011) aimed to determine if the common marmoset would be a viable large animal model of infection. A lethal infection was established with aerosol challenge doses below 10 CFU. Because of the extreme lethality, a precise LD_50_ was not able to be determined. They concluded that the common marmoset should be considered as a suitable model for further studies of inhalational melioidosis.

## Genetic Characterization

*Burkholderia pseudomallei* is a soil-dwelling saprophyte and with genetic diversity resulting from horizontal gene transfer (Tuanyok et al., 2008; Zhu et al., 2011). These transfers are recognized as regions in the genome [genomic island (GI) regions] with lower G/C content and that encode a broad array of functions. Genetic variability has been demonstrated in strains isolated from a single patient and isolates from different tissues within the patient (Hayden et al., 2012). This genetic variability makes it essential that the genomes of each strain in the panel be genetically characterized. *B. pseudomallei* strains have been genotyped using PCR methods, such as random amplified polymorphic DNA (RAPD; Leelayuwat et al., 2000) and multilocus sequence typing (MLST; Godoy et al., 2003; McCombie et al., 2006). RAPD detects differences in genomes by amplifying segments of unknown DNA. These PCR fragments are separated based upon electrophoretic mobility and the strains are compared by the presence or absence of bands. The production of PCR artifacts makes this genotyping method less robust than MLST genotyping. MLST compares the sequence of artificially concatenated amplicons of seven housekeeping genes. These genes are assumed to be selectively neutral or under purifying selection. MLST provides nucleotide data for multiple haplotypes, is amenable to phylogenetic analyses. Currie et al. (2007) reported that the MLST genotypes of strains from Australia were distinct from strains isolated in Thailand. Another PCR-based method variable number tandem repeat (VNTR) loci has been shown as a useful tool for genotyping *B. pseudomallei*. A VNTR locus consists of tandemly repeated sequences of DNA that vary in copy number, creating PCR amplicon size polymorphisms that are easily detected with gel electrophoresis. Due to increased mutation rates when compared to other regions of DNA and their multi-allelic nature, VNTRs allow superior discrimination between closely related isolates. Variations in VNTR can be observed in serially passed isolates (U’Ren et al., 2007; Price et al., 2010).

Finally, the rapid reduction in the cost of whole genome sequencing makes it feasible to compare the genomic sequence of different strains and to identify any genetic changes occurring due to *in vitro* or *in vivo* passage. Therefore, the scoring for genetic characterization was:

0 points for no genetic data (note that there are no criteria under which we assigned 1 or 2 points, given the importance of genetic characterization)
3 points for MLST
4 points for sequencing of GIs
5 points for sequenced genome data.

Twenty strains received the highest score in this rating system (Table 4) although a discussion of the features of the sequencing efforts is beyond the scope of this review.

**Table 4:** ***Burkholderia pseudomallei* strains with genome sequences**.

Bacterial strain	Whole genome sequenced data	MLST genotype	Genetic characterization score
K96243	http://www.ncbi.nlm.nih.gov/nuccore/NC_006350	Yes	5
MSHR296	http://www.mggen.nau.edu/BurkNextGenProj/BurkNextGenProj.html	Yes	5
MSHR840	http://www.mggen.nau.edu/BurkNextGenProj/BurkNextGenProj.html	Yes	5
RF43-BP22	http://www.mggen.nau.edu/BurkNextGenProj/BurkNextGenProj.html	Unknown	5
S191	P. Keim, personal communication	ST315	5
1106a	http://www.ncbi.nlm.nih.gov/nuccore/NC_009076	ST70	5
VN-Rat	P. Keim, personal communication	Unknown	5
1710b	http://www.ncbi.nlm.nih.gov/nuccore/NC_007434	ST177	5
TGH	P. Keim, personal communication	Unknown	5
W294	P. Keim, personal communication	Unknown	5
1106b	http://www.ncbi.nlm.nih.gov/bioproject/54361	ST70	5
Pasteur 52237	http://www.ncbi.nlm.nih.gov/bioproject/54289	ST411	5
S13	http://www.ncbi.nlm.nih.gov/bioproject/54287	ST51	5
1655	http://www.ncbi.nlm.nih.gov/bioproject/54283	ST131	5
1710a	http://www.ncbi.nlm.nih.gov/bioproject/54285	ST177	5
MSHR668	http://www.ncbi.nlm.nih.gov/genome/476?project_id = 58389	ST129	5
406e	http://www.ncbi.nlm.nih.gov/bioproject/54371	ST211	5
576	http://www.ncbi.nlm.nih.gov/bioproject/55425	ST501	5
MSHR346	http://www.ncbi.nlm.nih.gov/bioproject/55259	ST243	5
1026b	http://www.ncbi.nlm.nih.gov/nuccore/385657254 and http://www.ncbi.nlm.nih.gov/nuccore/CP002834	ST102	5

## Strain Availability

Because an overarching objective of our *B. pseudomallei* research is to find common animal models that are acceptable to the FDA for Animal Rule research, common, well-characterized strains would ideally be made available to the *B. pseudomallei* research community. Therefore, we reviewed the legal constraints for individual *B. pseudomallei* strains in publicly available data and personnel communications. Strains were assigned a score on a scale of zero through five using the following six criteria. While the grading scale used encompassed five through zero, no score of three was assigned to increase the distinction between restricted and unrestricted strains.

0 points for no information identified
1 point for license required for use or distribution
2 points for MTA required
4 points for strains available through a collaborator’s collection without restriction
5 points for strains available in the public domain.

Many of the strains we investigated were available, and we identified 52 strains available in the public domain as listed in Table 5. Further discussion of availability of strains is beyond the scope of this review.

**Table 5:** ***Burkholderia pseudomallei* strains available in the public domain**.

Bacterial strain	Availability	Intellectual property score
9	Public domain	5
112	Public domain	5
576	Public domain	5
1026b	Public domain	5
DD503	Public domain	5
DL2	Public domain	5
DL25	Public domain	5
DL28	Public domain	5
DL35	Public domain	5
DL36	Public domain	5
INT2-BP127	Public domain	5
INT2-BP190	Public domain	5
INT2-BP24	Public domain	5
INT2-BP270	Public domain	5
INT2-BP38	Public domain	5
INT2-BP91	Public domain	5
INT4-BP18	Public domain	5
K96243	Public domain	5
MSHR296	Public domain	5
MSHR305	Public domain	5
MSHR487	Public domain	5
MSHR503	Public domain	5
MSHR520	Public domain	5
MSHR644	Public domain	5
MSHR668	Public domain	5
MSHR730	Public domain	5
MSHR840	Public domain	5
NAU14B6	Public domain	5
NAU21B9	Public domain	5
NAU24B3	Public domain	5
NAU33A4	Public domain	5
NAU44A6	Public domain	5
NCTC 13178	Public domain	5
NCTC 13178(CL24)	Public domain	5
NCTC 13179	Public domain	5
NCTC 13179(CL07)	Public domain	5
Pasteur 52237	Public domain	5
PB08298010	Public domain	5
RF43-BP22	Public domain	5
RF67-BP1	Public domain	5
RF6-BP15	Public domain	5
RF85-BP37	Public domain	5
S141	Public domain	5
S191	Public domain	5
S241	Public domain	5
S43	Public domain	5
S90	Public domain	5

## Panel Selection and Discussion

With the overall objective of creating a panel of well-characterized *B. pseudomallei* strains for use in animal model development, we totaled the scores for strain source, passage history, virulence, genotype, phenotype, and availability for each strain. We then selected the six strains with the highest scores to develop into a panel of *B. pseudomallei* reference strains, with the intention of using these strains to develop animal models (Table 6). The selected strains were 1026b, MSHR668, K96243, MSHR305, 1106a, and 406e. MSHR668 was isolated from a human encephalomyelitis case. Strain 1026b has been used extensively in animal infection models of melioidosis, is available in the public domain, and has a published annotated genome sequence. Furthermore, 1026b was isolated from a human case without pulmonary involvement. However, a low passage stock of 1026b was not located. Nonetheless, the extensive animal research conducted with 1026b as well as an annotated genome sequence makes it a good choice for the reference panel. While it was not isolated from a pulmonary presentation case and had no animal virulence data, the relatively low passage number, availability of genome sequence data, and availability to laboratories made MSHR668 a good choice for the reference panel. Strain K96243 is a well-studied laboratory strain of *B. pseudomallei* isolated from a human case. While this strain was not isolated from a pulmonary case and had an unknown passage history, it has sequence data and extensive mouse virulence data, including studies indicating a very low LD_50_. In addition, K96243 is readily available, making it a good choice for a panel of *B. pseudomallei* strains for animal models of melioidosis. MSHR305 was also isolated from a human encephalomyelitis case, but with low passage, MLST data and some animal virulence data in mice, this strain also may prove useful in animal models of melioidosis. Strain 1106a was isolated from a human case but details on pulmonary presentation were not available. However, the 1106a genome is sequenced and the intratracheal infection of rats resulted in a lethal disease. Furthermore, a low passage isolate could be obtained, making 1106a a good candidate for inclusion on the panel. Finally, 406e was isolated from a human case with pulmonary involvement, genome sequence data, a low LD_50_ in intraperitoneal infection of hamsters and the availability of a relatively low passage isolate made 406e the highest scoring *B. pseudomallei* strain in our literature review and a very good candidate for inclusion in a panel of reference strains for animal models of melioidosis. With these data, it is our intention to develop a set of cell banks to serve as a reference panel of *B. pseudomallei* strains for use in animal models of melioidosis.

**Table 6:** **Characteristics of *B. pseudomallei* strain panel**.

Bacterial strain	Country of origin	Strain source/clinical outcome	Passage history/date of isolation	Molecular characterization	Animal model			Scoring system					
					LD_50_ or ID_50_ (CFU)	Route of infection	Species	Reported animal virulence data	Origin/isolation characterization	Genetic characterization	Minimum passage	Intellectual property/availability	Total
MSHR 668	Australia	53-Year-old male patient from Darwin with severe melioidosis encephalomyelitis; survived with good cognitive function but residual hemiparesis	6/1995	MLST and genome sequence	No data	No data	No data	0	4	5	4	5	18
MSHR 305	Australia	Brain of a fatal melioidosis encephalitis case	6/Unknown	MLST and genome sequence	Highly virulent	No data	Mouse	2	4	3	4	5	18
1026b	Thailand	Blood from 29-year-old female rice farmer with diabetes mellitus and disseminated disease including bacteremia, soft tissue, joint, and splenic involvement; survived to discharge	Unknown/1993	MLST only	10 to 4 × 10^4^ CFU	Aerosol, intraperitoneal, intratracheal, intranasal	Hamster, mouse, rat, guinea pig	5	4	5	0	5	19
1106a	Thailand	23-Year-old female rice farmer presenting to Sappasithiprasong Hospital, survived to discharge	8/1993	MLST and genome sequence	1.3 × 10^2^ CFU	Intratracheal	Rat	3	4	5	3	4	19
K96243	Thailand	34-Year-old female diabetic patient/Lethal	Unknown (>18)/1996	MLST and genome sequence	5 to 3 × 10^7^ CFU	Aerosol, intraperitoneal, intranasal, subcutaneous	Mouse, marmoset	5	4	5	0	5	19
406e	Thailand	Toe swab isolated from a 21-year-old male laborer with disseminated disease including bacteremia, lung, skin, and renal tract involvement; died	8/1988	MLST and genome sequence	<10	Intraperitoneal	Hamster	3	5	5	3	4	20

## Conflict of Interest Statement

The authors declare that the research was conducted in the absence of any commercial or financial relationships that could be construed as a potential conflict of interest.
